# Incidental finding of undiagnosed aortic coarctation in an elderly patient with a rare association with thymic carcinoma: a case report with review of the literature

**DOI:** 10.1016/j.radcr.2023.07.003

**Published:** 2023-07-12

**Authors:** Manar Ezzahi, Ayman Bijbij, Amal Akammar, Nizar El Bouardi, Meriem Haloua, Moulay Youssef Alaoui Lamrani, Meryem Boubbou, Mustapha Maaroufi, Badreeddine Alami

**Affiliations:** Department of Radiology and Interventional Imaging, CHU Hassan II Fez, Sidi Mohammed Ben Abdellah University, Fez, Morocco

**Keywords:** Aortic coarctation, Incidental finding, Thymic carcinoma

## Abstract

Aortic coarctation is a congenital malformation that is relatively prevalent, occurring in approximately 5 out of every 1000 births. The narrowing typically happens at the aortic isthmus between the left subclavian artery and the arterial ligament. It is frequently associated with a bicuspid aortic valve.

Generally, coarctation of the aorta is identified and treated during childhood or early adulthood. If left untreated, this condition can lead to a reduced life expectancy in individuals who have not received treatment.

We present a case of a 52-year-old man who complained of chest pain, sputum, and hemoptysis persisting for approximately 2 years. Contrast-enhanced computed tomography (CT) scans revealed the presence of an anterior mediastinal mass, which was later confirmed to be a thymic carcinoma (on histological study). Additionally, an incidental finding of a thoracic aortic coarctation with a well-developed collateral circulation was observed.

The discovery of aortic coarctation in adult patients as an incidental finding is rare and particularly uncommon in association with mediastinal or thoracic tumor pathology.

Adult and elderly patients with uncorrected coarctation generally have a low survival rate, and the management strategies for such cases are controversial, especially when it is associated to other pathologies.

Due to the complexity of therapeutic management and the limited literature available on postsurgical outcomes in these cases, making therapeutic decisions requires a multidisciplinary approach and personalized consideration for each individual case. This approach is necessary to evaluate the risk-benefit ratio and determine the most suitable therapeutic solution.

## Introduction

Aortic coarctation refers to a significant narrowing of the aortic lumen, leading to an obstruction of blood flow. This condition is relatively common and is notable for its diverse forms and the severity of its possible complications.

The severity, location, and extent of aortic coarctation can vary significantly among patients and depend on the age at which the condition is diagnosed. Traditionally, Bonnet's classification differentiated postductal coarctation found in older children and adults from preductal coarctation observed in infants and newborns. However, with modern investigative techniques, a clinical distinction without preconceptions about the anatomy appears to be more appropriate.

The diagnosis of aortic coarctation primarily depends on clinical evaluation and can be made in 2 main situations. In over 50% of cases, coarctation is diagnosed before the first year of life when a newborn or infant presents symptoms of cardiac distress that can significantly affect their vital prognosis, leading to suspicion of coarctation. On the other hand, there are other forms of coarctation that typically remain asymptomatic and are incidentally detected at various ages, including infancy, childhood, or even adulthood, without immediate indications of severity.

The discovery of this condition in elderly patients remains infrequent, particularly when it is associated with other regional pathologies as observed in our case. As a result, it presents challenges in terms of diagnosis, assessing associated malformations, and determining the impact on treatment decisions.

## Case presentation

We present a case of a 52-year-old male with no prior medical or surgical history. The patient has a 35-year history of chronic cigarette smoking, but quit 6 months ago. He has been experiencing chest pain, dyspnea at stage II of the Modified Medical Research Council (mMRC) scale, sputum production, and hemoptysis for the past 2 years. There are no other respiratory or nonrespiratory symptoms present, and the patient's overall health and body temperature remain stable.

Six months prior to admission, the patient's dyspnea worsened, reaching stage III on the mMRC scale with apparition of chest pain. The patient's cough also worsened, accompanied by the production of mucous sputum, leading him to seek medical attention at the hospital.

During the physical examination, the patient's temperature was 36.2°C (oral), blood pressure measured 137/88 mm Hg in the right arm and 125/84 mm Hg in the left arm, the pulse rate was 78 beats per minute, and the respiratory rate was 18 breaths per minute. The respiratory and cardiovascular examination yielded normal findings.

Laboratory tests including white blood cell count, platelet count, c-reactive protein, serum electrolytes, and urine analysis were normal.

**Fig. 1:**
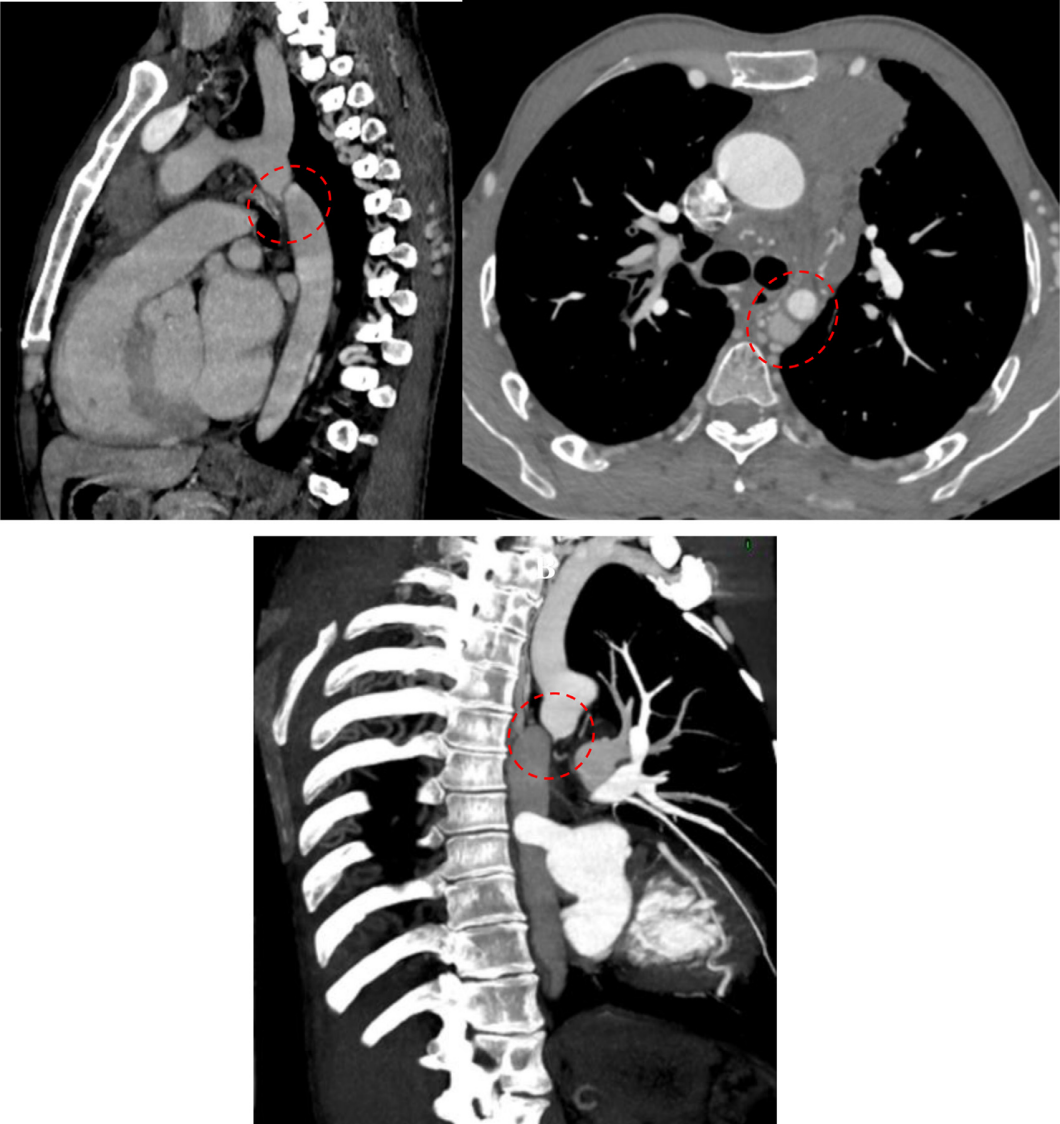
Chest CT angiography with sagittal and axial reconstructions. Critical (red circle) focal stenosis of the thoracic aorta of isthmic location, related to coarctation of the aorta, the site of coarctation is 47 mm from the left subclavian artery.

**Fig. 2:**
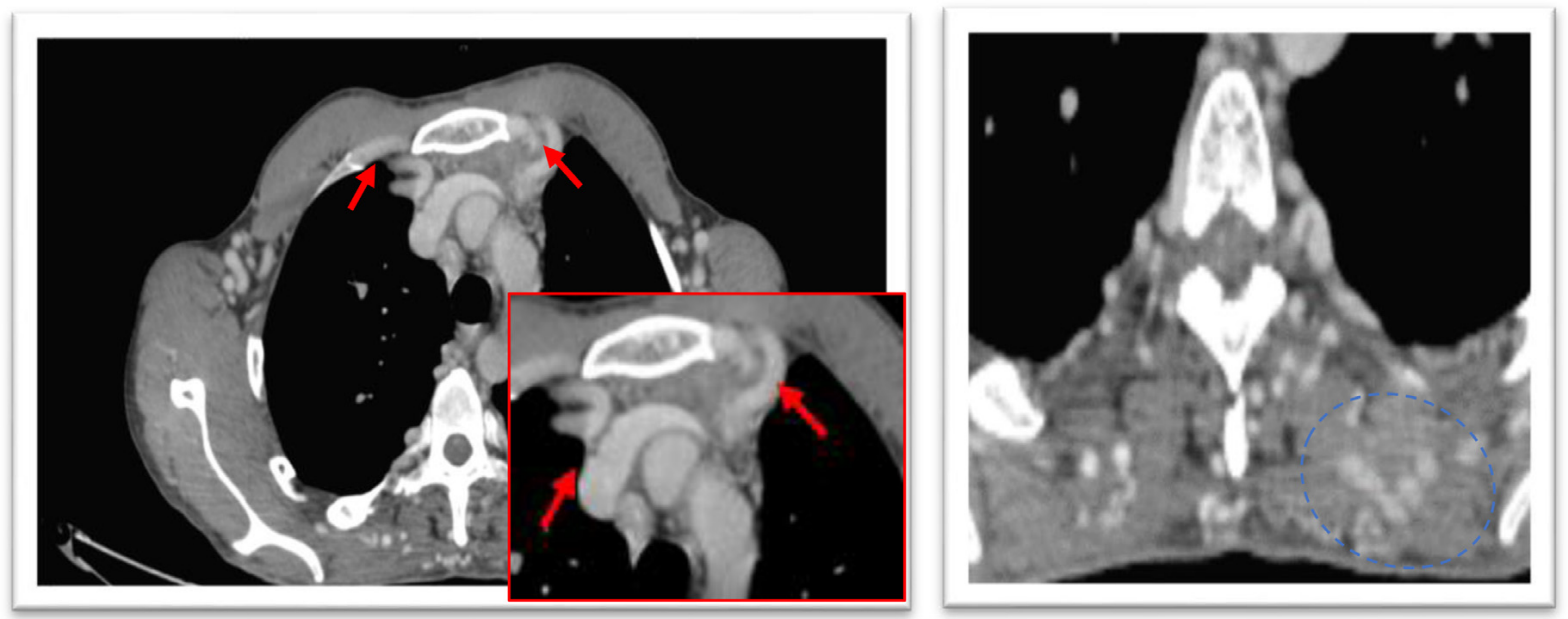
Axial-enhanced thoracic CT scan showing: Extensive collateral arterial circulation involving the intercostal, internal mammary (red arrow), bronchial, epigastric, and posterior chest wall (blue circle) muscle plane arteries which are dilated. Provides a bypass route for the poststenotic descending aorta with recovery of a satisfactory caliber downstream measured at 21 mm poststenotic and 13 mm (abdominal suprarenal aorta).

**Fig. 3:**
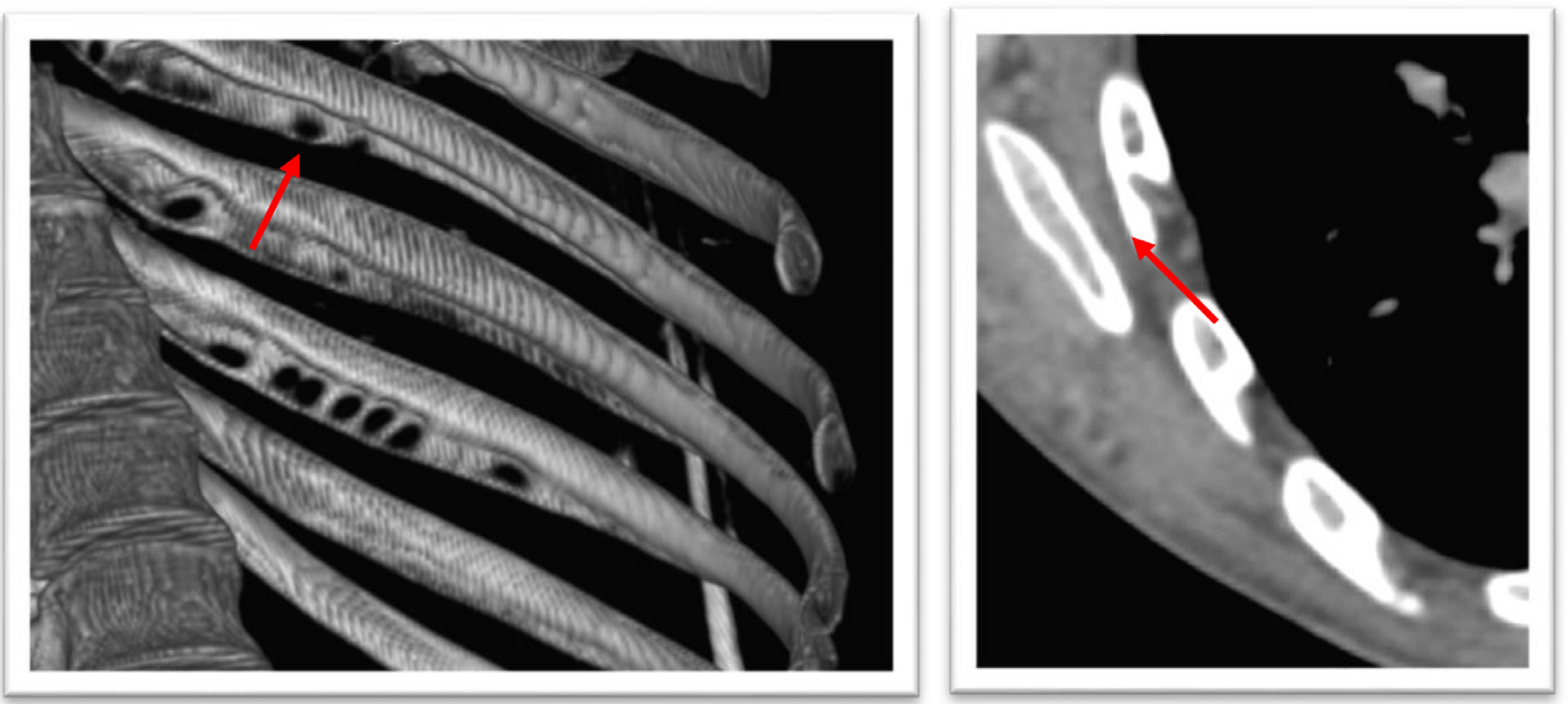
CT-scan 3D reconstruction: Inferior rib notching (red arrow): Roesler sign, secondary to dilated intercostal collateral vessels which form as a way to bypass the coarctation and supply the descending aorta. • The dilated and tortuous vessels erode the inferior margins of the ribs, resulting in notching.

**Fig. 4:**
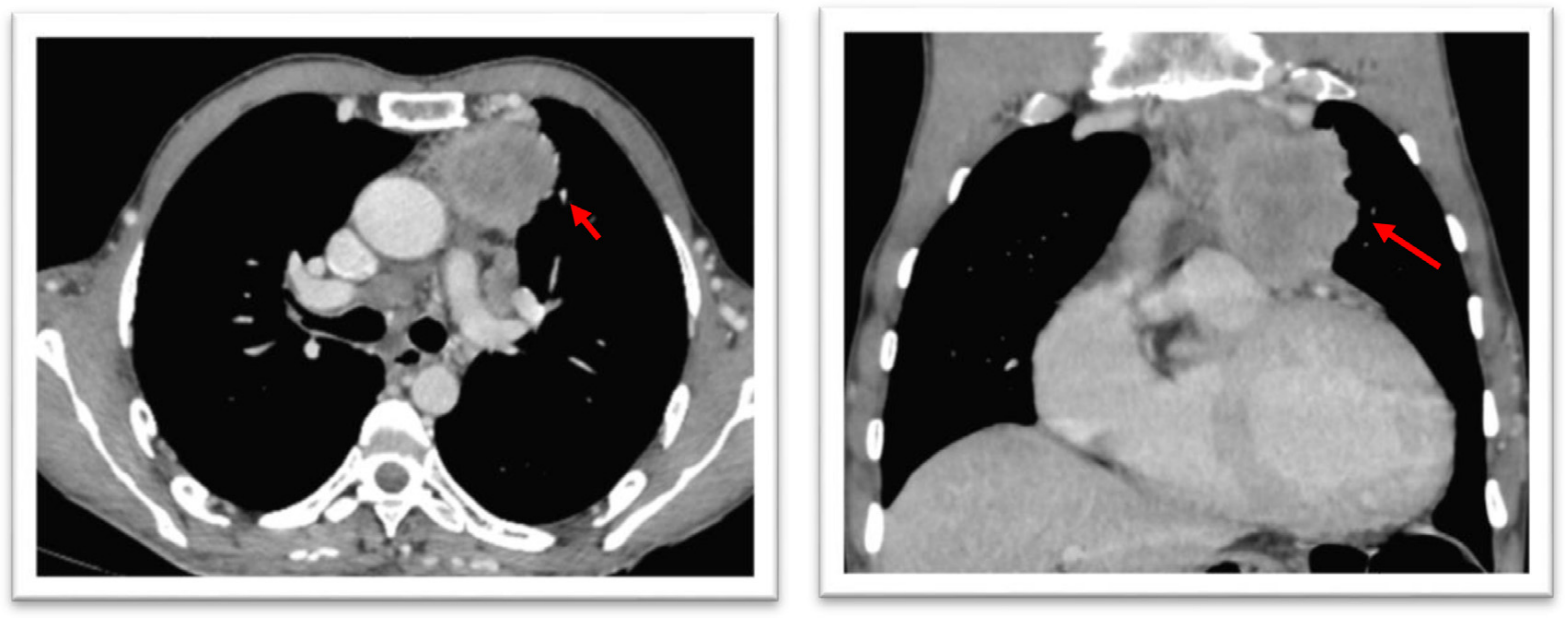
Axial-enhanced thoracic CT scan (A) and coronal reconstruction (B) showing: Locally advanced thymic carcinoma stage III of Masoaka system, well limited with poly-lobed contours, heterogeneously enhanced after contrast, containing intratumor necrosis. • Reaching aortic arch superiorly and on the trunk of the pulmonary artery and part of the right ventricle inferiorly, which are permeable and of normal caliber, with loss of the fatty separation border. • Externally: It invades the left mediastinal pleura and bulges toward the left upper lobe (red arrow).

A contrast-enhanced thoracoabdominal CT scan was performed, which revealed an isthmic aortic coarctation (Fig. 1) with extensive collateral circulation (Fig. 2) compensating for the restricted blood flow downstream in the descending aorta. A 3D reconstruction of the CT scan showed the characteristic “notching” sign on the inferior ribs, indicating the presence of dilated intercostal collateral vessels that form as a bypass to supply the descending aorta in response to the coarctation (Fig. 3). The scan also identified a locally advanced anterior mediastinal tumor (Fig. 4).

A CT-guided biopsy was conducted to obtain a tissue sample from the anterior mediastinal mass. The histological examination, along with immuno-histochemical study (IHC), confirmed the presence of thymic carcinoma.

A transthoracic echocardiography (TTE) was also performed, which revealed findings of ischemic cardiomyopathy, characterized by an impaired left ventricular function with a left ventricular ejection fraction (LVEF) of 20%. Additionally, anterosepto-apical hypokinesia was observed. No other associations or abnormalities were noted during the examination.

Unfortunately, due to the locally advanced stage of the tumor, the extensive collateral circulation and the patient's age; the patient was deemed ineligible for a surgical intervention plan. The presence of extensive collaterality poses additional challenges to the surgical procedure, making it more difficult to achieve successful outcomes.

As a result, the patient's heart condition is being managed medically, and preventive measures have been implemented.

## Discussion

Aortic coarctation is a congenital malformation of the aorta that should be diagnosed and corrected early in life. Unrepaired coarctation of the aorta leads to the development of systemic hypertension, increased morbidity, and death from cardiovascular disease. If the condition is left uncorrected, most patients die by the fifth decade of life, and very few survive in older age. The age at correction has been demonstrated as the most important factor for the relief of hypertension and long-term survival [1].

In literature, there are few reports of patients who has been first diagnosed with uncorrected coarctation at very late age (>60-70 years), and there is no clear consensus on how to manage them.

Survival until 52 years old is exceptional in patients with uncorrected coarctation since coronary artery disease is a significant cause of early death in these patients. In our case, TTE demonstrates ischemic cardiomyopathy with a low LVEF of 20% and anterosepto-apical hypokinesis.

The presence of this malformative disease, in our patient, made a veritable challenge in the surgical decision and management of his thymic carcinoma. The surgical team judged that the surgical intervention is too risky for this patient, given the locally advanced character of the thymic tumor and in other hand the extensive regional vascular collateral channels secondary to severe and evolutive aspect of his aortic coarctation discovered lately. This case illustrates the complexity of the therapeutic management in these rare cases, where a mediastinal or thoracic lesion or process coexists with a severe coarctation of the aorta. However, the absence of systemic hypertension and relatively low transaortic gradient with well-developed collateral vessels, together with the patient's unwillingness to undergo further treatment, led us to be conservative concerning his CoA.

Despite that, surgical management is an independent beneficial prognostic factor for thymic tumors, and all patients with thymic tumors should seek surgical treatment as soon as possible [2]. In our case, we think that this would have carried far too much perioperative risk. It is doubtful whether the correction would have prolonged the patient's survival or improved his quality of life.

Transcatheter balloon dilation with or without stent implantation is now being performed increasingly for treatment of coarctation. It is recommended actually as the therapy of choice in experienced centers [3,4]. Favorable postsurgical results have been reported in adolescents and adults with aortic coarctation [4], but experience and evidence of benefit in elder patients still scarce.

### Imaging features

Aortic coarctation can be primarily divided into 2 types:

→ Infantile (preductal) form: characterized by a narrowing of the aorta from the distal part of the brachiocephalic artery to the level of the ductus arteriosus, with typically a more discrete area of constriction just upstream of the ductus but downstream of the origin of the left subclavian artery. Therefore, the blood supply to the descending aorta is via the persistence of the ductus arteriosus.
→ Adult form (juxta-ductal, postductal, or middle aortic): Characterized by abrupt stenosis of short segment of the postductal aorta. It is due to thickening of the aortic media and usually occurs just downstream of the ligamentum arteriosum.

### Chest radiography

Despite low sensitivity, chest radiography can raise suspicion for CoA [5].

Leftward convexity of the descending aorta with enlargement of the left subclavian artery may be seen in children and young adults with CoA, as well as a “Figure 3” sign formed by prestenotic and poststenotic dilatation of the descending aorta. In older patients, notching of the posterior ribs, from the fourth to the eighth, related to collateral blood flow in the enlarged intercostal arteries may be seen. The notching of posterior ribs is seen only in long-standing cases, and therefore not seen in infancy (unusual in patients <5 years of age). If the coarctation is distal to either subclavian artery, then increased flow occurs through the subclavian artery, forming a collateral pathway via the internal thoracic artery, anterior intercostal artery, posterior intercostal artery, and then into the descending thoracic aorta.

### Transesophageal echocardiography and transthoracic echocardiography

They are the first imaging modalities for suspected CoA, given their easier availability and safety. They could provide hemodynamic parameters such as the CoA-gradient using Doppler [6], and can assess cardiac function and associated cardiac and valvular abnormalities [7]. The visualization of the CoA-site can be difficult due to a poor acoustic window [8]. TTE also has limited value in the evaluation of extracardiac structures and collateral circulation [6].

### Computed tomography

This imaging tool has a high spatial resolution of both intracardiac and extracardiac structures and allows 2-dimensional and 3-dimensional reconstructions of relevant vascular anatomy.

The main disadvantage of CT is the cumulative radiation dose from repeated examinations, especially in the pediatric population [5].

The introduction of multidetector CT with iterative reconstructions has significantly reduced radiation doses to values substantially below 1 mSv [8].

Furthermore, current state-of-the-art CT scans can obtain the entire volumetric data acquisition in a single or several cardiac cycles, which minimize motion artifacts and can eliminate the requirement for breath-holding [8].

### Cardiac magnetic resonance imaging

Cardiac magnetic resonance imaging (CMR) is the preferred advanced imaging modality for diagnosis and follow-up [9]. The advantage is the absence of ionizing radiation, making it perfect for follow-ups. Three-dimensional gadolinium-enhanced CMR angiography provides a good assessment of aortic morphology, location and degree of stenosis, and the extent of collateral vessel formation [9]. It is important to note that measurement of aortic dimensions alone may not be sufficient to assess the degree of severity of CoA, as the hemodynamic effect of CoA is influenced by a complex interplay between aortic geometry, vessel wall mechanics, flow, and ventricular function [10]. Cine imaging allows analysis of left ventricular function and myocardial mass, while phase contrast flow analysis allows estimation of the pressure gradient across the CoA and calculation of collateral flow. The new 4-dimensional flow CMR provides an assessment of flow velocities and pressure fields along the aorta, the pressure gradient across a CoA, wall shear stress (WSS), oscillatory shear index (OSI) can be calculated using computational fluid dynamics. Despite these advantages, CMR is hampered by the long acquisition time and the need to hold the breath during the scan, which limits its use in small children and claustrophobic patients [9]. Sedation or general anesthesia may be required. In addition, metal stenting causes CMR artifacts, which limits adequate follow-up evaluation [9].

## Conclusion

Coarctation of the aorta is rarely diagnosed in patients of advanced age. Elderly patients with uncorrected coarctation should be examined and treated for possible complications of coarctation, such as hypertension, coronary artery disease, and cerebral aneurysms. In our situation, the surgical solution has been discarded, given the complexity of the pathological association, and the increased risk in the perioperative period. Especially since no similar case has been found in the literature that proves the real benefit of invasive treatment. Given the rarity of these cases and the lack of consensus, the therapeutic approach must be multidisciplinary and must be personalized for each case, in order to assess the risk-benefit ratio and promote the best therapeutic solution.

## Patient consent

Written informed consent was obtained from the patient, and legal guardian for publication of this case report and any accompanying images. A copy of the written consent is available for review by the Editor-in-Chief of this journal.
